# Cytokines and Exhaled Nitric Oxide Are Risk Factors in Preterm Infants for Bronchopulmonary Dysplasia

**DOI:** 10.1155/2021/6648208

**Published:** 2021-01-12

**Authors:** Zhenjie Zhang, Wuchen Wu, Lian Hou, Jingjing Jiang, Weilin Wan, Zhenghong Li

**Affiliations:** ^1^Department of Pediatrics, Peking Union Medical College Hospital, Chinese Academy of Medical Sciences, Beijing, China; ^2^Department of Neurosurgery, Shenzhen University General Hospital, Shenzhen, China; ^3^Department of Clinical Lab, Peking Union Medical College Hospital, Chinese Academy of Medical Sciences, Beijing, China

## Abstract

Bronchopulmonary dysplasia (BPD) is the most common complication of extremely preterm birth. This study was aimed at detecting cytokine and fractional exhaled nitric oxide (FeNO) levels to evaluate their mechanisms and predicted significance for BPD. Preterm infants born at *gestational* *age* ≤ 32 *weeks* were recruited, and clinical data were collected. We detected ten cytokines, including IFN-*γ*, IL-10, IL-12p70, IL-13, IL-1*β*, IL-2, IL-4, IL-6, IL-8, and TNF-*α* on Days 1–3, Days 7–14, and Days 21–28 after birth by using the Meso Scale Discovery (MSD) technology. The FeNO levels of infants were measured when they met the discharge criteria. A total of 46 preterm infants were enrolled, consisting of 14 infants in BPD group and 32 infants in the control group. The gestational age (27.5 ± 1.3 vs. 29.9 ± 1.3 weeks) and birth weight (1021 ± 261 g vs. 1489 ± 357 g) were lower in the BPD group. The following were high-risk factors for BPD, as determined by multivariate logistic regression analysis: *gestational* *age* < 30 *weeks*, *birth* *weight* < 1000 *g*, PDA, longer mechanical ventilation, and higher FeNO. The cytokines of IL-6 and IL-8 on Days 7–14 and IL-4, IL-6, IL-8, and TNF-*α* on Days 21–28 were also high-risk factors for BPD. IL-6 contributed to BPD disease severity. *Conclusion*. The preterm infants with PDA and prolonged mechanical ventilation tended to develop BPD. The IL-6 and IL-8 were significantly increased on Days 7–14 and were high-risk factors for BPD. Moreover, the IL-6 level was associated with BPD disease severity. We speculated that NO was related to BPD via Th2 cell-mediated inflammatory responses such as IL-4 and IL-6. Cytokines might predict the occurrence of BPD.

## 1. Introduction

Bronchopulmonary dysplasia (BPD) is the most frequently reported complication of extremely preterm birth. BPD is currently regarded as a result of an aberrant respiratory distress response to the developing lungs. Mechanical ventilation and long-term use of oxygen may contribute to the onset of BPD [1]. However, despite multiple strategies to provide ventilation and oxygen therapy, the incidence of BPD has not decreased. Significant advances in the development of animal BPD models have been made. Regardless, clinical research still needs to be conducted to explore the pathogenesis and treatment of BPD.

In preterm infants, episodes of sepsis and the ongoing use of supplemental oxygen, mechanical ventilation, and parenteral nutrition have been associated with systemic inflammatory responses. Specifically, these are associated with the effects of inflammatory cytokines and chemokines. IL-6, IL-8, IL-10, and TNF-*α* are significantly altered in tracheal aspirate samples in infants with BPD [2]. This research represents an advanced study on inflammation and explores the risk attributed to selected inflammatory cytokines (IFN-*γ*, IL-10, IL-12p70, IL-13, IL-1*β*, IL-2, IL-4, IL-6, IL-8, and TNF-*α*) in venous blood samples from preterm infants.

Fractional exhaled nitric oxide (FeNO), one of the most high-potential biomarkers for airway inflammation, is well related to eosinophilic airway inflammation. In 1993, a study was conducted to detect airway inflammation by measuring FeNO levels, which was found to have a significant positive correlation to the severity of inflammation [3]. In 2009, the American Thoracic Society (ATS)/European Respiratory Society (ERS) joint statement affirmed the clinical application of FeNO as a biological indicator of airway inflammation [4]. In 2011, ATS published clinical guidelines for FeNO [5]. FeNO testing is simple, reliable, and reproducible, rendering it ideal for noninvasive evaluation of airway inflammatory diseases. Therefore, the current study targeted preterm infants to detect cytokines and FeNO to evaluate their mechanism and possible predicted significance for BPD.

## 2. Methods

We conducted a prospective study, which was approved by the Ethics Institutional Review Board of Peking Union Medical College Hospital. Signed informed consent was obtained from the parents of each infant admitted to the Neonatal Intensive Care Unit (NICU) who were agreeable to the use of residual serum from routine testing for cytokine detection.

### 2.1. Study Population

*Inclusion criteria*: (1) preterm infants born at gestational age ≤ 32 weeks, (2) preterm infants born from January 2018 to October 2019, (3) preterm infants admitted to the NICU at Peking Union Medical College Hospital within 3 days from birth, and (4) the parents signed the informed consent.

*Exclusion criteria*: (1) infants with severe congenital malformations, (2) infants with severe infection or shock, (3) infants with inherited metabolic diseases, (4) infants who died, discharged, or transported to other hospitals before their discharge criteria were met.

Infants diagnosed with BPD were assigned to the BPD group, whereas infants without BPD were assigned to the control group. BPD was diagnosed as oxygen dependency > 28 d after birth, as defined at a workshop by the National Institutes of Child Health; the Human Development/National Heart, Lung, and Blood Institute; and the Office of Rare Diseases [6]. The inhaled oxygen concentration of 21% on Day 28 was divided into the mild group, and the inhaled oxygen concentration of 21-30% was divided into the moderate group. No infants were diagnosed with severe BPD in the study.

### 2.2. Data Collection and Analysis

Clinical data were prospectively collected by one researcher and verified by another. Baseline data on infant characteristics were collected, including gestational age, birth weight, Apgar score, neonatal respiratory distress syndrome (NRDS), anemia, patent ductus arteriosus (PDA), eosinophils, duration of mechanical ventilation (in days) (MV), duration of invasive ventilation (in days), and so on. Eosinophilia was defined as absolute eosinophil count greater than 700/mm^3^ [7]. The baseline data of the maternal characteristics were collected, including delivery mode, antenatal steroids, and premature rupture of membrane (PROM), among others.

### 2.3. Inflammation Biomarkers

To avoid a sample selection bias, the parents or legal guardians of eligible infants were approached as early as possible after birth (usually <72 h). To limit invasiveness, the research was analyzed only from the residual (leftover) serum of weekly routine testing from the hospital laboratory. Serum cytokines were detected on Days 1–3, Days 7–14, and Days 21–28 after birth. Our study detected ten cytokines, including IFN-*γ*, IL-10, IL-12p70, IL-13, IL-1*β*, IL-2, IL-4, IL-6, IL-8, and TNF-*α*. To minimize protein degradation, strict protocols were employed in our hospital laboratory. Blood collected in BD Microtainers (Becton Dickinson, Mississauga, Ontario, Canada) was spun down within 1 h to remove the cell fraction, and the leftover serum was immediately stored at −80°C. The serum was collected to detect the cytokine protein level via the MSD technology using the U-PLEX Biomarker Group 1 (Human) Multiplex Assay (K15049D). A MESO QuickPlex SQ 120 machine and MSD Discovery Workbench version 4.0.12 were used for detection and data analysis, respectively.

### 2.4. Detection of FeNO Levels

FeNO levels in infants were measured when discharge criteria were met (weight > 2000 g, corrected gestational age > 34 w, and full oral feeding). FeNO was measured with the offline tidal breathing method according to the ATS/ERS recommendations for standardized procedures for exhaled lower respiratory nitric oxide [8]. No bronchodilators or nebulized inhaled hormones were used 12 h before the test. Before FeNO samples were obtained, samples of ambient NO in the room were collected. Measurements were determined when ambient NO was below 5 parts per billion (ppb). Adequate room ventilation was assured. Exhaled air can be collected via a facemask connected to a non-rebreathing valve that allows inspiration of NO-free air from an NO-inert reservoir to avoid contamination by ambient NO. Exhaled breath samples are collected into an NO-inert bag fitted with the expiratory port once a stable breathing pattern is present. The expiratory port of the valve provides an expiratory resistance. This resistance will only help to avoid nasal contamination if the mask does not cover the nose. Analyses were conducted immediately after collection. FeNO was measured using the NIOX Flex (Aerocrine, Sweden) by ozone-/NO_2_-based chemiluminescence detection. Calibrations were performed following manufacturer instructions. The average of three measurements for each infant was taken.

### 2.5. Statistics

Statistical analysis was conducted using SPSS 25.0. Normally distributed quantitative data were presented by the mean ± standard error ($\bar{X}\pm S$). Comparison of data between two groups was conducted using the *t*-test. The quantitative data that failed to meet the normal distribution was described by *M* (P25-P75), and comparison between groups was tested using the rank sum test. The qualitative data were described by frequency and rate. Pearson's chi-squared test or Fisher's exact test was conducted. Logistic regression was used to analyze the independent risk factors of BPD. *P* < 0.05 was considered statistically significant.

## 3. Results

### 3.1. Baseline Data

From January 2018 to October 2019, 52 preterm infants were born at gestational age ≤ 32 weeks. Four preterm infants were excluded because of insufficient residual serum after routine testing for cytokine detection. Two preterm infants died from severe infection and thus were excluded. A total of 46 preterm infants were enrolled in this study, including 14 infants in the BPD group and 32 infants in the control group. Nine BPD patients were diagnosed with mild disease and 5 BPD patients with moderate disease. A total of 138 blood samples were collected. The incidence of BPD was 30%. The gestational age (27.5 ± 1.3 vs. 29.9 ± 1.3 weeks, *P* = 0.000) and birth weight (1021 ± 261 g vs. 1489 ± 357 g, *P* = 0.000) of the BPD group were lower than those of the control group. As shown in Tables 1-3, the incidence rates of PDA, anemia, and NRDS were higher in the BPD group; moreover, the values of the duration of mechanical ventilation, parenteral nutrition, and length of hospital stay were significantly higher in the BPD group.

### 3.2. Inflammatory Cytokine Profile of BPD Patients

The number of cytokine data points over time confirmed the similar distribution between infants with or without BPD in the first 4 weeks. A total of 1380 times of cytokines were assessed from 138 blood samples. The resulting ten cytokines include IFN-*γ*, IL-10, IL-12p70, IL-13, IL-1*β*, IL-2, IL-4, IL-6, IL-8, and TNF-*α*, as shown in Table 4. On Days 1–3, IL-10 was significantly decreased in the BPD group; on Days 7–14, IL-6, IL-8, and TNF-*α* were significantly increased in the BPD group, whereas IL-10 was decreased. On Days 21–28, IL-1*β*, IL-4, IL-6, IL-8, and TNF-*α* were significantly increased in the BPD group, whereas IL-10 was decreased.

### 3.3. Comparison of FeNO and Eosinophils between Two Groups

All enrolled preterm infants were tested for FeNO before discharge; there was no significant difference in the corrected gestational age on the testing day (36.4 ± 3.2 and 35.2 ± 2.3 weeks, *t* = 1.407, *P* = 0.169) between the two groups. The FeNO level was higher in the BPD group than in the control group (13.9 ± 6.2 and 8.2 ± 3.3 ppb, *t* = 2.967, *P* = 0.006), and the differences were statistically significant Table 3. Eosinophilia in the BPD group was significantly increased 21–28 d from birth, and the differences were statistically significant (*P* values = 0.043).

### 3.4. Multivariate Logistic Regression of BPD Influencing Factors

Multivariate logistic regression analysis was conducted in this study. Statistically significant factors in univariate analysis were used as independent variables, whereas BPD was used as the dependent variable (Table 5). The data indicated that gestational age < 30 weeks, birth weight < 1000 g, PDA, and longer duration of mechanical ventilation were high-risk factors for BPD. The cytokines IL-6 and IL-8 on Days 7–14, as well as IL-4, IL-6, IL-8, and TNF-*α* on Days 21–28, were also high-risk factors for BPD. Other risk factors for BPD included a high FeNO level.

### 3.5. IL-6 Contributed to BPD Disease Severity

We observed increased IL-6 and IL-8 levels on Days 7–14 in BPD infants. Nine BPD patients with mild disease and 5 BPD patients with moderate disease were analyzed in this study. IL-8 level was not significantly different between mild and moderate BPD infants (196.4 ± 93.2 and 255.2 ± 90.3, *P* = 0.769). Compared to mild BPD infants, IL-6 was significantly increased in moderate BPD infants (6.4 ± 3.5 and 12.2 ± 9.3, *P* = 0.034). In the 7-14 day samples, IL-6, but not IL-8, contributed to BPD disease severity.

## 4. Discussion

The survival rates of extremely-low-birth-weight infant (ELBWI) and very-low-birth-weight infant (VLBWI) have increased in recent years, and the incidence of BPD has rapidly risen. ELBWI/VLBWI indicated extreme immaturity due to the structure and function of the lung. Various damage factors, such as oxides, free radicals, hypoxia, infection, ventilator pressure, volume damage, or alveolar shear stress, among others, directly cause the release of inflammatory factors via proinflammatory factors, chemokines, and proteases, leading to BPD.

Gestational age and birth weight are the most important factors affecting the occurrence of BPD [9]. Other known risk factors for BPD included maternal preeclampsia [10], chorioamnionitis [11], sepsis [12] and fetal growth restriction [13], and early mechanical ventilation > 7 d [14]. In the present study, birth gestational age < 30 weeks, birth weight < 1000 g, and prolonged durations of mechanical ventilation were risk factors for BPD, as determined by multivariate analysis.

Our study determined that PDA was a high-risk factor for BPD, which we attributed to the ability of PDA to prolong the duration of mechanical ventilation after birth. Severe left-to-right shunts in PDA infants could cause pulmonary congestion and edema. Long-term left-to-right shunts could aggravate both pulmonary vascular damage and pulmonary inflammation. Thus, by minimizing the duration of mechanical ventilation, actively closing the arterial catheter, comprehensive treatment, and standardized management can potentially reduce the incidence and risk of BPD to improve the prognosis of preterm infants.

Both prenatal and postnatal inflammation could cause an imbalance between proinflammatory and anti-inflammatory factors in the body. A large number of proinflammatory factors were released, exacerbating damage to the immature lung and causing BPD. This result was verified by previous animal experiments. In a previous study, IL-6 was involved in a murine hyperoxia model of BPD [15]. This finding was consistent with the results of clinical trials. In BPD infants, IL-1*β*, IL-6, TNF-*α*, and IL-10 cytokine levels were altered in the amniotic fluid [16], cord blood [17], and tracheal aspirate samples [2]. TNF-*α* single nucleotide polymorphisms could predict BPD onset and severity in preterm neonates [18]. Ehrhardt et al. reported that TNF-*α* in tracheal aspirate samples was associated with BPD severity [19]. We thus speculated that these cytokines could directly affect lung injury that could lead to BPD in premature infants. We found that the cytokines IL-6 and IL-8 on Days 7–14, as well as IL-4, IL-6, IL-8, and TNF-*α* on Days 21–28, were high-risk factors for BPD. Our data exhibited potential for clinical application. Our results demonstrated dynamic changes in cytokines at different time points. Some cytokines (e.g., IL-4, IL-6, IL-8, and TNF-*α*) showed no significant changes in the early stages but exhibited significant changes in the late stages.

It is also possible that changes in some of the serum cytokines reflect the magnitude of lung injury, with more severe lung injury resulting in greater cytokine concentrations. Some cytokine responses may be attempts to compensate or attenuate the lung injury. IL-10 could be a protective factor against the development of BPD. IL-10 can reduce acute lung injury secondary to hyperoxia by inhibiting inflammation, reducing acute lung injury caused by mechanical stretching [20]. In the study of normal macrophage culture in vitro, it was found that IL-10 can inhibit the production of endotoxin-induced oxygen free radicals and reactive oxygen species, inhibit the activation of nuclear transcription factor-*κ*B (NF-*κ*B), and thus reduce the expression of proinflammatory cytokines such as IL-8 [21]. Exogenous IL-10 downregulates the inflammatory mediators of high mobility group box 1 protein. The expression level reduces the level of inflammatory factors such as TNF-*α*, thereby inhibiting the progression of inflammation, improving the cell infiltration of lung tissues, thereby producing a significant lung protective effect.

The cytokines IL-6 and IL-8 on Days 7–14 were high-risk factors for BPD. IL-8 is produced by many types of leukocyte as well as fibroblasts, endothelial cells, and epithelial cells in response to other cytokines, bacterial or viral products, and environmental stressors (e.g., reactive oxygen intermediates). In adults with acute lung injury, IL-8 levels correlate with mortality and morbidity, and a greater reduction in plasma IL-8 is noted with smaller tidal volumes, indicating that volutrauma may be involved in IL-8 release [22]. IL-8 strongly attracts neutrophils, and antibodies to IL-8 or its receptor reduce neutrophil influx and lung injury in various experimental models. IL-6 is a proinflammatory cytokine that mediates acute lung injury, exacerbates ventilator-induced lung injury, and plays an essential role in lung inflammation in preterm newborns [23]. IL-6 is produced by stimulated monocytes, fibroblasts, and endothelial cells and has multiple biological activities. IL-6 is produced at sites of inflammation and plays a key role in the transition from acute to chronic inflammation.

The IL-6 and IL-8 were significantly increased on Days 7–14 and were high-risk factors for BPD. Moreover, IL-6 level was associated with BPD disease severity. The cytokines could predict the occurrence of BPD to a certain extent. Sustained systemic inflammation in infants prior to clinical symptoms of BPD suggested that anti-inflammatory interventions can be more effective in the early than late stages.

Bronchial hyperreactivity is common in preterm infants with BPD and is likely due to airway damage in early infancy caused by intubation and MV. Besides arresting lung maturation, lung inflammation is a major contributor to the pathogenesis of BPD. Since lung inflammation may be caused primarily by oxygen and/or MV, there is increased interest in postnatal measurements of exhaled NO, a marker of eosinophilic airway inflammation [24, 25]. Animal studies have shown that at lower concentrations, NO has a bronchodilator effect, but at higher levels, NO has a proinflammatory action. The increased levels of NO observed in disease states caused by airway inflammation are thought to be the result of increased induction of nitric oxide synthase and of increased reactive nitrosylation pathways [26]. May et al. showed that the levels of exhaled nitric oxide were increased in infants with established BPD, particularly in those developing moderate or severe BPD, and could indicate ongoing inflammation [27]. Our study found that FeNO and eosinophil levels were higher in the BPD group than in the non-BPD group. Multivariate analysis identified FeNO as a risk factor for BPD. The results indicated that BPD was closely correlated to eosinophilic airway inflammation.

NO is generated by NO synthase (NOS). The three NOS isoforms are endothelial NOS (eNOS), neuronal NOS (nNOS), and inducible NOS (iNOS). NO and IL-4 affect and promote each other. In previous research, a mouse model lacking all NOS isoforms (n/i/eNOS-/-) was used to elucidate the authentic role of NO/NOSs. Pathological findings of the n/i/eNOS-/- mice revealed significant decreases in bronchial eosinophilic inflammation, bronchial thickening, mucus secretion, and mRNA levels of IL-4, IL-5, and IL-13. These results indicated that NO played an important role in promoting eosinophilic inflammation and mucus hypersecretion that might be related to an increase in Th2 cytokine such as IL-4, IL-5, and IL-13 [28]. Meanwhile, IL-4 could also increase the expression of iNOS [29]. IL-4 is known to induce the alternative activation pathway in M2 macrophages, which regulate NO production by inducing arginase I. Arginase I catalyzes the hydrolysis of L-arginine, a common substrate of iNOS, thereby regulating NO synthesis by competing with the substrate [30]. The expression of iNOS can be observed in epithelial, endothelial, neural, and smooth muscle cells, eosinophils, neutrophils, and alveolar macrophages in the lung after its induction by IL-4 and/or IL-13 [31, 32]. Immunity develops gradually during gestation, and there is a significant immaturity of the cellular immune response at birth. In the present study, both IL-4 and FeNO were increased and related to BPD, as determined by univariate analysis and multifactor analysis. Our exploratory analyses indicate that infants who develop BPD have elevated IL-8 accompanied by a relative predominance of Th2 cytokines (IL-4 and IL-6). We speculated that NO was related to BPD via Th2 cell-mediated inflammatory responses such as IL-4 and IL-6. However, excessive NO was reported to frequently induce immune and pathological processes and mediate the production of some inflammatory mediators, resulting in tissue damage [33].

One limitation of this study is that serum cytokine concentrations may not accurately reflect the cytokine concentrations in the lung; moreover, the target cells in the lung that produce or respond to the cytokines cannot be identified. However, such limitation also exists in the case of tracheal aspirates or bronchoalveolar lavage, both of which reflect the epithelial lining fluid rather than the alveolar or interstitial milieu. Another limitation of the study is the small sample size. We could not further assign BPD infants to different groups by their severity. Further studies with a larger sample size are required to assess the value of cytokines and FeNO in the diagnosis of BPD.

## 5. Conclusion

BPD is a disease resulting from a combination of multiple factors. It is closely related to eosinophilic airway inflammation. Preterm infants with PDA, prolonged mechanical ventilation, and high FeNO level showed a tendency to develop BPD. The cytokines of IL-6 and IL-8 on Days 7–14 and IL-4, IL-6, IL-8, and TNF-*α* on Days 21–28 were high-risk factors for BPD. IL-6 contributed to BPD disease severity. The cytokines may predict the occurrence of BPD to a certain extent. Our study speculated that NO was related to BPD via Th2 cell-mediated inflammatory responses such as IL-4 and IL-6.

## Figures and Tables

**Table 1:** The basic infant characteristics between the BPD and non-BPD groups.

	BPD group (*n* = 14)	Non-BPD group (*n* = 32)	*P* value
Male gender: *n* (%)	9 (64.3%)	16 (50%)	0.370
Singleton: *n* (%)	10 (71.4%)	28 (87.5%)	0.183
Birth weight: $\bar{X}\pm S$ (grams)	1021 ± 261	1489 ± 357	0.000
Gestational age: $\bar{X}\pm S$ (weeks)	27.5 ± 1.3	29.9 ± 1.3	0.000
Small gestational age (SGA): *n* (%)	0	1 (3.1%)	1.000
Apgar 1 min: $\bar{X}\pm S$	7.5 ± 2.5	8.4 ± 1.3	0.140
Apgar 5 min: $\bar{X}\pm S$	8.9 ± 1.5	9.5 ± 0.8	0.090
Apgar 10 min: $\bar{X}\pm S$	9.3 ± 0.9	9.6 ± 0.6	0.084
NRDS: *n* (%)	14 (100%)	23 (71.9%)	0.048
Patent ductus arteriosus (PDA): *n* (%)	12 (85.7%)	12 (37.5%)	0.004
Late-onset neonatal sepsis (LOS): *n* (%)	7 (50%)	15 (46.9%)	0.844
Anemia: *n* (%)	14 (100%)	23 (71.9%)	0.030
Minimum hemoglobin: $\bar{X}\pm S$ (g/l)	101 ± 8.7	105 ± 12.9	0.220
Duration of mechanical ventilation (days): *M* (P25-P75)	36 (27-53)	10 (7-15)	0.000
Duration of invasive ventilation (days): *M* (P25-P75)	4 (2.7-7.2)	0	0.000
Nebulized inhaled hormone and bronchodilator: *n* (%)	8 (57.1%)	4 (12.5%)	0.003
Intravenous hormone: *n* (%)	4 (28.6%)	2 (6.3%)	0.060
Duration of parenteral nutrition: $\bar{X}\pm S$ (days)	42.1 ± 12.1	25.6 ± 10.2	0.000
Length of hospital stay: $\bar{X}\pm S$ (days)	58.6 ± 17.6	34.0 ± 11.1	0.000
Corrected gestational age at discharge: $\bar{X}\pm S$	36.2 ± 1.2	34.6 ± 1.0	0.000

*N* represents the number of patients.

**Table 2:** The basic maternal characteristics between the BPD and non-BPD groups.

	BPD group (*n* = 14)	Non-BPD group (*n* = 32)	*P* value
Maternal age: $\bar{X}\pm S$ (years)	30.7 ± 3.5	33.5 ± 4.1	0.065
Caesarean section: *n* (%)	10 (71.4%)	29 (90.6%)	0.173
Antenatal steroids: *n* (%)	10 (71.4%)	17 (53.1%)	0.246
Premature rupture of membranes (PROM): *n* (%)	5 (35.7%)	17 (53.1%)	0.023
Intrauterine infection: *n* (%)	2 (14.3%)	7 (21.9%)	0.700

*N* represents the number of patients.

**Table 3:** The comparison of FeNO and eosinophilia between the BPD and non-BPD groups.

	BPD group (*n* = 14)	Non-BPD group (*n* = 32)	*P* value
D1-3 eosinophilia: *n* (%)	3 (21.4%)	0 (0%)	0.050
D7-14 eosinophilia: *n* (%)	2 (14.3%)	6 (18.7%)	1.000
D21-28 eosinophilia: *n* (%)	7 (50%)	12 (37%)	0.043
FeNO: ppb	13.9 ± 6.2	8.2 ± 3.3	0.006

*N* represents the number of patients with increased eosinophils.

**Table 4:** The cytokines of the patients.

	BPD group (*n* = 14)	Non-BPD group (*n* = 32)	*P* value
Cytokines (pg/ml) *M* (P25-P75) Days 1-3			
IFN-*γ*	8.7 (6.8-18)	8.7 (5.8-14.0)	0.582
IL-10	2.6 (1.8-4.6)	3.1 (1.3-3.8)	0.043
IL-12p70	1.2 (0.45-2.2)	0.6 (0.4-0.9)	0.454
IL-13	5.7 (5.1-11.1)	7.2 (4.7-10.9)	0.896
IL-1*β*	1.5 (0.9-14.1)	1.4 (0.9-9.2)	0.940
IL-2	2.7 (1.0-4.1)	1.7 (1.0-3.8)	0.680
IL-4	0.33 (0.2-0.7)	0.25 (0.1-0.6)	0.217
IL-6	6.6 (3.8-7.4)	7.4 (3.8-12.9)	0.830
IL-8	176 (137-370)	159 (108-271)	0.895
TNF-*α*	0.5 (0.3-1.1)	0.20 (0.1-1.2)	0.370
Cytokines (pg/ml) *M* (P25-P75) Days 7-14			
IFN-*γ*	21.1 (4.2-26.2)	11.5 (9.1-17.6)	0.721
IL-10	2.4 (1.6-8.1)	3.2 (1.1-7.4)	0.036
IL-12p70	0.79 (0.36-2.51)	0.58 (0.38-1.15)	0.897
IL-13	5.8 (4.7-9.3)	5.2 (4.0-8.8)	0.537
IL-1*β*	1.95 (0.78-2.35)	1.5 (0.82-2.74)	0.062
IL-2	2.28 (0.76-6.54)	1.39 (0.66-3.46)	0.516
IL-4	0.43 (0.11-1.63)	0.27 (0.14-0.92)	0.673
IL-6	7.91 (2.90-22.03)	5.2 (3.1-8.1)	0.033
IL-8	240.9 (89.7-363.3)	126 (87-339)	0.001
TNF-*α*	0.9 (0.2-2.1)	0.4 (0.2-2.9)	0.007
Cytokines (pg/ml) *M* (P25-P75) Days 21-28			
IFN-*γ*	9.8 (5.6-17.3)	17.4 (11.3-26.2)	0. 150
IL-10	2.3 (2.1-4.3)	2.9 (2.25-4.41)	0.008
IL-12p70	0.67 (0.41-1.01)	0.90 (0.53-1.14)	0.426
IL-13	7.2 (4.5-10.4)	9.1 (4.6-10.7)	0.488
IL-1*β*	2.86 (1.69-3.77)	2.15 (0.58-3.31)	0.042
IL-2	1.87 (0.87-2.09)	1.94 (0.74-2.98)	0.739
IL-4	0.44 (0.12-1.18)	0.20 (0.085-1.18)	0.002
IL-6	9.52 (2.34-5.81)	5.45 (2.27-13.0)	0.036
IL-8	459 (116-301)	255.7 (69.3-499.6)	0.005
TNF-*α*	1.2 (0.98-1.78)	0.6 (0.2-1.5)	0.002

**Table 5:** Multivariate logistic regression of BPD influencing factors.

	OR	95% CI	*P* value
Gestational age < 30 weeks	7.472	3.021-17.325	0.000
Birth weight < 1000 g	11.265	3.134-24.325	0.000
PDA	5.388	2.216-11.103	0.000
Duration of mechanical ventilation	1.893	1.342-2.768	0.001
FeNO	2.547	1.397-4.824	0.000
D7-14 IL-6	1.133	1.008-1.451	0.026
D7-14 IL-8	1.092	1.021-1.438	0.031
D21-28 IL-4	1.245	1.127-2.824	0.000
D21-28 IL-6	1.276	1.091-1.671	0.001
D21-28 IL-8	1.325	1.146-1.872	0.020
D21-28 TNF-*α*	1.219	1.105-1.584	0.025

## Data Availability

The data used to support the findings of this study are available from the corresponding author upon request.

## Abbreviations

ATS: American Thoracic Society
BPD: Bronchopulmonary dysplasia
ERS: European Respiratory Society
FeNO: Fractional exhaled nitric oxide
NRDS: Neonatal respiratory distress syndrome
PDA: Patent ductus arteriosus.
